# A New Broach

**Published:** 1881-02

**Authors:** 


### A NEW BROACH.

Dr. C. R. Butler, of Cleveland, has devised and made an improved broach for excising and removing the pulp tissue from the roots of teeth; one that seems better adapted for the purpose than anything else in use.

It is made of the best quality of piano wire. Two or three sizes are used. They are dressed to a triangular form and brought to a sharp point, and are given a slight curvature, to facilitate the passage into irregular canals. They are sharply barbed, and, when properly introduced and manipulated, will excise and remove all pulp tissue from any ordinary canal.

They are called "Butler's Excising Broaches," and will, probably, ere long be in the market.

